# Screening for malaria antigen and anti-malarial IgG antibody in forcibly-displaced Myanmar nationals: Cox’s Bazar district, Bangladesh, 2018

**DOI:** 10.1186/s12936-020-03199-4

**Published:** 2020-03-30

**Authors:** Austin Lu, Olivia Cote, Silvia D. Dimitrova, Gretchen Cooley, A. Alamgir, M. Salim Uzzaman, Meerjady Sabrina Flora, Yulia Widiati, Mohammad Saifuddin Akhtar, Maya Vandenent, Daniel C. Ehlman, Sarah D. Bennett, Leora R. Feldstein, Eric Rogier

**Affiliations:** 1 Georgia State University, Atlanta, GA 30302 USA; 2 Georgia Institute of Technology, Atlanta, GA 30332 USA; 3 Division of Parasitic Diseases and Malaria, Centers for Disease Control and Prevention, Atlanta, GA 30329 USA; 4 Synergy America, Inc., Duluth, GA 30097 USA; 5 Institute of Epidemiology, Disease Control and Research, Dhaka, Bangladesh; 6 United Nations Children’s Fund, Motel Road, Cox’s Bazar, 4700 Bangladesh; 7 United Nations Children’s Fund, 1 Minto Road, Dhaka, 1000 Bangladesh; 8 Global Immunization Division, Center for Global Health, U.S. Centers for Disease Control and Prevention, Atlanta, GA 30329 USA; 9 Epidemic Intelligence Service, Centers for Disease Control and Prevention, Atlanta, GA 30329 USA

**Keywords:** Multiplex serology, Antigen detection, Malaria, *P. falciparum*, *P. malariae*, *P. vivax*

## Abstract

**Background:**

Several refugee settlements in Bangladesh have provided housing and medical care for the forcibly-displaced Myanmar nationals (FDMN, also known as Rohingya) population. The identification of malaria infection status in the refugee settlements is useful in treating infected persons and in developing malaria prevention recommendations. Assays for *Plasmodium* antigens and human IgG against *Plasmodium* parasites can be used as indicators to determine malaria infection status and exposure.

**Methods:**

Dried blood spot (DBS) samples (N = 1239) from a household survey performed April–May 2018 in three settlements in Cox’s Bazar district, Bangladesh were utilized for a sample population of children from ages 1–14 years of age. The samples were tested using a bead-based multiplex antigen assay for presence of the pan-*Plasmodium* antigen aldolase as well as *Plasmodium falciparum* histidine rich protein 2 (HRP2). A bead-based multiplex assay was also used to measure human IgG antibody response to *P. falciparum, Plasmodium malariae,* and *Plasmodium vivax* merozoite surface protein 1 antigen (MSP1) isoforms, and *P. falciparum* antigens LSA1, CSP, and GLURP-R0.

**Results:**

There were no detectable *Plasmodium* antigens in any samples, suggesting no active malaria parasite infections in the tested children. IgG seroprevalence was highest to *P. vivax* (3.1%), but this was not significantly different from the percentages of children antibody responses to *P. falciparum* (2.1%) and *P. malariae* (1.8%). The likelihood of an anti-*Plasmodium* IgG response increased with age for all three malaria species. Evidence of exposure to any malaria species was highest for children residing 8–10 months in the settlements, and was lower for children arriving before and after this period of time.

**Conclusions:**

Absence of *Plasmodium* antigen in this population provides evidence that children in these three Bangladeshi refugee settlements did not have malaria at time of sampling. Higher rates of anti-malarial IgG carriage from children who were leaving Myanmar during the malaria high-transmission season indicate these migrant populations were likely at increased risk of malaria exposure during their transit.

## Background

Malaria is transmitted by the female Anopheles mosquito and remains a significant public health concern with approximately 216 million cases globally and 445,000 deaths annually in 2016 [1]. Parasites belonging to the genus *Plasmodium* are the causative agents of malaria, and a significant burden to humanity with nearly half of the human population at risk for infection. Children are particularly vulnerable, in part because they have not yet developed protective immunity [2], and in 2016, it was estimated that approximately two-thirds of global malaria mortality was from children under the age of five [3].

In dealing with *Plasmodium* infections, the human immune system has adapted to recognize numerous *Plasmodium* antigens to be targeted for humoral response [4, 5]. Individuals with high levels of parasite-specific antibodies lower susceptibility to *Plasmodium falciparum* infection and morbidity [6]. The *Plasmodium* merozoite surface protein 1 (MSP1) antigen has been well characterized in immunological studies, and is known to induce “long-lived” IgG responses [7], with species-specific isoforms eliciting specific responses with limited cross-reactivity [8]. *Plasmodium* exposure even at a young age has the potential to induce a decades-long, or even life-long, antibody response. Specifically for *P. falciparum*, numerous antigens are known to induce more “short-lived” IgG responses, an presence of IgG against these antigens indicates more recent *P. falciparum* exposure [7, 9, 10].

In 2006, Bangladesh had approximately 3 million malaria cases with 26,000 deaths [11], but by 2017, fewer than 5000 malaria cases were reported, with the majority of remaining transmission occurring in the Chittagong division [1]. Malaria is endemic within 13 districts located in the southeast and northeast regions of the country, and highly seasonal [12]. In the Cox’s Bazar district, located within the Chittagong division, *P. falciparum* is thought to cause over 70% of malaria cases [1, 13]. Directly to the southeast of Cox’s Bazar, the Myanmar states of Rakhine and Chin represent some of the highest malaria transmission zones in Myanmar [1]. Many settlements of persons fleeing violence in Myanmar are currently located in this malaria epidemic border region [14]. With the influx of more than 727,000 forcibly-displaced Myanmar national (FDMN) refugees into Cox’s Bazar since August 2015 [15], the detection of malaria exposure and active infection status is vital for understanding the malaria status and transmission dynamics within the refugee settlements. Populations living in both the formal and makeshift camps are at increased risk to many infectious diseases due to overcrowding, lack of sanitation, and poor sewage disposal [16]. The World Health Organization is collaborating with the government of Bangladesh to use malaria rapid diagnostic tests (RDT) to provide fast and accurate diagnosis of malaria, and meet any health needs of the community within the settlements [17, 18].

To determine the prevalence of active infection and assess for past malaria exposures in this refugee population, samples were tested from children living in three camps in Bangladesh near the Myanmar border. As of mid-year 2018 (when the survey took place), it was estimated that almost 650,000 persons live among these three settlements [18]. Multiplex bead assays (MBAs) were used to test for presence of malaria antigens as well as IgG antibodies against *P. falciparum, Plasmodium vivax*, and *Plasmodium malariae*.

## Methods

### Ethics approval and consent to participate

The survey was approved by the Bangladesh Institute of Epidemiology, Disease Control and Research institutional review board; and was approved at the US Centers for Disease Control and Prevention (CDC) as a non-research surveillance activity. All persons participating in the study were assigned a 6-digit identification number that could not be traced back to the individual. Participation was voluntary, and the objectives, participation time, and risks/benefits of participation were explained to each participant prior to collecting data. Verbal informed consent was obtained and the caregiver of the child answered questions of a short survey including the child’s age and duration in the camp. The initial survey design was powered to measure childhood vaccination coverage and estimate immune protection status through IgG titers to vaccine preventable disease antigens, and those results will be published elsewhere. Simple random sampling of households took place in the formal camps (Nayapara and Kutupalong) and a multi-stage cluster household sampling in the makeshift settlements. In each selected household, one child 6 months to 6 years of age and one child aged 7 years to 14 years was randomly selected. When designing the study, the expected number of children for total enrollment was 1089 children aged 6 months–6 years and 1055 children aged 7 to 14 years. In a subset of children (approximately 914 children aged 1 to 6 years of age and 787 children aged 7 to 14 years) 3 to 4 drops of blood were expected for collection by finger prick.

### Sample collection

Following a finger prick using 1.8-mm lancet, 3–4 drops of the child’s blood was placed on Whatman™ 903 Protein Saver Cards (GE Healthcare) and allowed to dry for at least 4 h to create a dried blood spot (DBS). A barcode of the identification number was affixed, and the samples were sealed in individual, disposable plastic bags containing silica gel desiccant and a humidity indicator card to protect them from humidity. The samples were transported to the CDC laboratories in Atlanta, GA for further testing and were stored at − 20 °C upon arrival and for long-term storage. Sample collection in Nayapara took place during Ramadan and many children were expected to observe fasting; as a result, DBS were not collected from 7 to 14-year-olds in that location. In Kutupalong Registered Camp, sampling was stopped early based on preferences of camp leadership.

### Blood elution and multiplex bead assays

A 3 mm DBS was punched from each card using a Harris Uni-Core Puncher. Each 3 mm DBS was eluted in 125 µL Elution Buffer (PBS, 0.05% sodium azide, 0.3% Tween-20, filtered with a 0.2 µm filter) overnight at 4 °C in a polystyrene round bottom 96-well plate (Costar^®^, Corning). It was assumed each 3 mm DBS contained 1.25 µL of serum (50% hematocrit); therefore, to perform a 1:100 dilution of the serum, the blood spot was eluted in a final volume of 125 µL. The DBS elutions were diluted 1:4 by adding 75 µL of the elution into 225 µL Buffer B (PBS, 0.5% polyvinyl alcohol, 0.8% polyvinylpyrrolidone, 0.5% casein, 0.3% Tween-20, 0.02% sodium azide, filtered with a 0.2 µm filter and containing 3 µg/mL crude *Escherichia coli* extract) for a final serum concentration of 1:400 used for the IgG detection assay. For the antigen detection assay, a 6 mm DBS punch was taken (10 µL whole blood), and whole blood eluted in the same manner as described above to a final dilution of 1:20 whole blood used for the assay.

Assays for *Plasmodium* antigen detection were performed as described previously [19]. Two unique bead regions (Bio-Plex COOH bead, BioRad, Hercules, CA) were individually coated by the EDC/Sulfo-NHS intermediate reaction with separate antibodies specific for each antigen to be captured: *Plasmodium* aldolase (12.5 µg/12.5 × 10^6^ beads, rabbit IgG anti-aldolase, Abcam, Cambridge, UK) and *P. falciparum* PfHRP2 (20 μg/12.5 × 10^6^ beads, mouse IgG anti-HRP2, Abcam). For the assay, a mix of the two anti-*Plasmodium* coupled bead regions was made in 5 mL Buffer A (PBS, 0.5% bovine serum albumin (BSA), 0.05% Tween20, 0.02% NaN_3_) so that 1500 of each bead region would be added per well in the assay plate. Samples at 1:20 dilution of whole blood were incubated with 50 µL of the bead mix in 0.2 µm filter bottom plates (Millipore) for 90 min under gentle shaking and subsequently washed three times with 100 µL wash buffer (PBS, 0.05% Tween20: PBS-T). Beads were incubated for 45 min with a 50 µL mix of detection antibodies: anti-pAldo (1:1000×, rabbit anti-aldolase, Abcam), and anti-HRP2 (1:500× of 1:1 mix of mouse IgG:IgM anti-HRP2, Abcam). All detection antibodies were previously biotinylated by Thermo Scientific EZ-Link Micro Sulfo-NHS-Biotinylation Kit (ThermoFisher Scientific) according to the manufacturer’s protocol. Plates were washed three times, and subsequently incubated with streptavidin-R phycoerythrin (1:200×, Invitrogen, Carlsbad, CA). Plates were washed three times, and after a final 30 min wash step with reagent diluent, beads were washed once and resuspended in 100 μL PBS and read on a Bio-Plex 200 instrument (BioRad, Hercules, CA**)** by generating the median fluorescence intensity (MFI) signal for a minimum of 35 beads in each unique region, and then the mean fluorescence intensity of the MFIs between duplicates. Positive and negative controls were included on each plate to ensure appropriate validity of data. The final measure, denoted as MFI-bg, was reported by subtracting MFI values from beads on each plate only exposed to sample diluent during the sample incubation step.

Six *Plasmodium* antigens were used for IgG antibody detection: the species-specific merozoite surface protein 19kD (MSP1) fragments for *P. falciparum*, *P. vivax*, and *P. malariae* (as described previously [8]), and three other *P. falciparum* antigens: liver stage antigen 1 (LSA1, [20]), circumsporozoite protein (CSP, NANPx5 repeat, [20]), and glutamate-rich protein R0 fragment (GLURP-R0, [21]). The three *Plasmodium* MSP1 19 kD antigens were produced recombinantly and purified as described previously [8], and the three other *P. falciparum* antigens were produced as peptides. The *Schistosoma japonicum* glutathione-*S*-transferase antigen was produced recombinantly and served as a generic protein to assess IgG non-specific binding. All antigens were coupled to magnetic beads (Luminex Corporation, Austin, TX) in the same manner as prior studies [8, 22]. Briefly, beads were pulse vortexed, transferred to a microcentrifuge tube and centrifuged for 1.5 min at 13,000*g*. Supernatant was removed and beads were washed with 0.1 M sodium phosphate, pH 6.2 (NaP). Beads were activated by suspending in NaP with 5 mg/mL of EDC (1-ethyl-3-[3′-dimethylaminopropyl]carbodiimide hydrochloride) and 5 mg/mL sulfo-NHS (sulfo *N*-hydroxysulfosuccinimide) and incubating with rotation for 20 min at room temperature (RT) protected from light. After a wash with coupling buffer (50 mM 2-(4-morpholino)-ethane sulfonic acid, 0.85% NaCl at pH 5.0), antigens were coupled to beads in presence of coupling buffer for 2 h at an antigen concentration of 20 µg/mL for all antigens except for CSP and GLURP-R0 at 30 µg/mL and GST at 15 µg/mL. Beads were washed once with PBS, and suspended in PBS with 1% BSA with incubation for 30 min at RT by rotation. Beads were then resuspended in storage buffer (PBS, 1% BSA, 0.02% NaN_3_, 0.05% Tween-20, protease inhibitors) and stored at 4 °C.

For the IgG assay, all incubation steps were performed at RT in 50 µL reaction volumes protected from light as previously described [23]. Incubation steps were followed by washes with 200 µL PBS-T performed by attaching plates to a handheld magnet (Luminex Corp., Austin, TX) for 1 min then inverting to evacuate supernatant. A master mix of beads was made by combining 125,000 beads per region in 5.5 mL of Buffer A. Bead master mix was aliquoted into a black flat-bottom 96-well plate (BioRad) for a total of 1250 beads per well per region. Beads were then incubated with eluted samples for 90 min and washed three times, followed by 45 min with secondary antibodies diluted in Buffer A (50 ng/well biotinylated mouse anti-human IgG and 20 ng/well biotinylated mouse anti-human IgG4; Southern Biotech, Birmingham, AL). After three washes, wells were incubated with 250 ng/well of streptavidin-R phycoerythrin, incubated for 30 min, washed three times, and incubated for 30 min with Buffer A alone. After one wash, beads were resuspended in 100 µL PBS and then stored overnight at 4 °C protected from light and read the following day. Before reading, plates were shaken at room temperature for at least 20 min, and the old PBS was removed. Fresh 100 µL PBS was added and plates were read on a MAGPIX machine (Luminex Corp) with Luminex xPonent^®^ software. Median fluorescence intensity (MFI) signal was generated for a minimum of 35 beads/region, and background MFI from wells incubated with Buffer B was subtracted from each sample to give a final value of MFI-bg.

### Statistical analysis

To determine an assay signal (MFI-bg signal) which would indicate positivity to antigen or antibody, a panel of 86 US resident blood samples were run by both the multiplex antigen assay (at 1:20 whole blood dilution) and multiplex antibody assay (at 1:200 whole blood dilution, equivalent to 1:400 serum) to obtain the mean and standard deviation for a “malaria non-exposed” population. For each antigen or antibody, the mean + 3 standard deviations (s.d). MFI-bg value for this non-exposed population was used at the positivity threshold. For antigen detection, the MFI-bg thresholds were: pAldo (325) and HRP2 (100), which correspond to a limit of quantification of approximately 128 pg/mL for pAldolase and 8.8 pg/mL for HRP2. For the IgG antibody detection assay, the MFI-bg thresholds were: PfMSP1 (75), PvMSP1 (146), PmMSP1 (207), LSA1 (99), CSP (120), GLURP-R0 (62). Positivity of a sample to any of the three *P. falciparum*-specific antigens (LSA1, CSP, GLURP-R0) considered that child positive to “Pf short-term” antibodies.

To obtain unweighted odds ratios (ORs) for IgG carriage to *Plasmodium* antigens, data was fit to logistic regression model in SAS v9.4 (Cary, NC) using PROC LOGISTIC with the MODEL statement. Wald OR estimates were obtained for camp of residence, age category, or duration at camp of residence. Duration categories were selected based on the seasonality of malaria for this region. Statistical significance was considered if 95% confidence interval did not include 1.0.

## Results

**Fig. 1:**
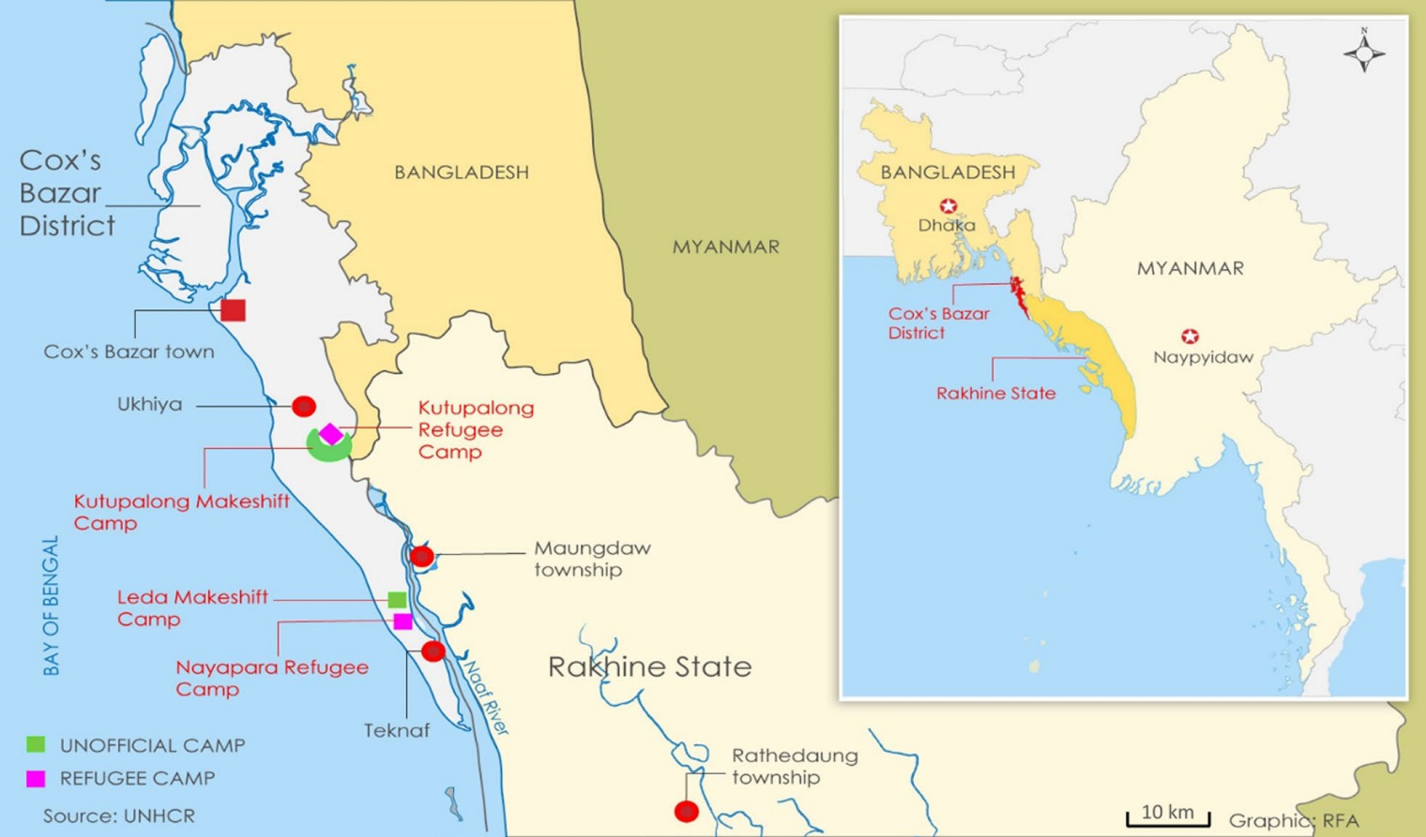
Locations of Myanmar refugee settlements in southeastern Bangladesh. In total, 1239 children were enrolled from the Nayapara (n = 273), Kutupalong (n = 309), and Kutupalong Makeshift Settlements (n = 657)

Figure 1 shows the location of the three refugee settlements included in this survey and number of children (N = 1239 total) providing a blood sample from each camp: Nayapara Registered Refugee Camp (henceforth referred to as Nayapara, n = 273), Kutupalong Registered Refugee Camp (henceforth referred to as Kutupalong, n = 309), and Kutupalong Makeshift Settlements (n = 657). In Table 1, seroprevalence of IgG to each antigen is reported by refugee camp, age category, and duration of residence at the camp in Bangladesh. None of the samples was positive for any *Plasmodium* antigens. Overall seroprevalence was low to each malaria species’ MSP1 antigen: *P. falciparum* at 2.1%, *P. vivax* at 3.1%, and *P. malariae* at 1.8%, and not statistically different from each other. None of the camp populations had a seroprevalence to any of the three species’ MSP1 antigens of greater than 3.9%. When generating seroprevalence estimates for any antibodies to any malaria species, the overall study population had an estimated seroprevalence of 7.3% with no single camp having a seroprevalence to any anti-malaria IgG above 8.9%.

**Table 1:** Prevalence of malaria antigen or anti-malaria IgG antibodies in 1239 Myanmar refugees residing in Bangladesh, April–May 2018

	IgG to *Plasmodium* antigen					
	PmMSP1 n/N (%)	PvMSP1 n/N (%)	PfMSP1 n/N (%)	Pf short-term n/N (%)	Any *Plasmodium* n/N (%)	*Plasmodium* antigen
Camp						
Nayapara	2/273 (0.7%)	5/273 (1.8%)	3/273 (1.1%)	2/273 (0.7%)	11/273 (4.0%)	0/273 (0.0%)
Kutupalong	7/309 (2.3%)	12/309 (3.9%)	4/309 (1.3%)	1/309 (0.3%)	22/309 (7.1%)	0/309 (0.0%)
Makeshift	13/657 (2.0%)	22/657 (3.4%)	19/657 (2.9%)	10/657 (1.5%)	57/657 (8.7%)	0/657 (0.0%)
Age (years)						
1–4	13/567 (2.3%)	15/567 (2.7%)	6/567 (1.1%)	4/567 (0.7%)	34/576 (6.0%)	0/567 (0.0%)
5–10	2/431 (0.5%)	12/431 (2.8%)	6/431 (1.4%)	4/431 (0.9%)	23/431 (5.3%)	0/431 (0.0%)
11–14	7/241 (2.9%)	12/241 (5.0%)	14/241 (5.8%)	5/241 (2.1%)	33/241 (13.7%)	0/241 (0.0%)
How long in Bangladesh						
1–7 months	0/10 (0.0%)	0/10 (0.0%)	0/10 (0.0%)	0/10 (0.0%)	0/10 (0.0%)	0/10 (0.0%)
8–10 months	13/640 (2.0%)	23/640 (3.4%)	18/640 (2.8%)	11/640 (1.7%)	57/640 (8.9%)	0/640 (0.0%)
> 10 months	9/587 (1.5%)	16/587 (2.7%)	8/587 (1.4%)	2/587 (0.3%)	33/587 (5.6%)	0/587 (0.0%)

Table 2 shows OR estimates for each subcategory with statistical significance indicated. The only significant difference observed in seroprevalence among the three settlements was seen in the Kutupalong in comparison to the Nayapara for IgG against any of the three *Plasmodium* species. Prevalence of IgG reliably increased with increasing age, but was only found to be statistically higher for the oldest age category (when compared to the youngest category) for the PfMSP1 and for antibodies against any malaria categories. Interestingly, the 5–10 year age category was significantly lower for PmMSP1 IgG in comparison to the 1–4 year age category. None of the 10 children residing in Bangladesh for less than 8 months was found to have IgG antibodies to malaria and, thus, OR regression estimates could not be generated for this group. Children residing in Bangladesh for 8–10 months were found to have higher seroprevalence to any species (and all species) in comparison to children residing in Bangladesh for more than 10 months, but this was only statistically significant for the categories of IgG to Pf short-term antigens, and IgG to any *Plasmodium* antigens.

**Table 2:** Odds ratio estimates (and 95% CIs) for IgG positivity to different *Plasmodium* antigens by resident camp, age, and duration in Bangladesh, among Myanmar refugees residing in Bangladesh, April–May 2018

	PmMSP1 OR (95% CI)	PvMSP1 OR (95% CI)	PfMSP1 OR (95% CI)	Pf short-term OR (95% CI)	Any *Plasmodium* OR (95% CI)
Camp					
Nayapara (referent)	1.0	1.0	1.0	1.0	1.0
Kutupalong	3.1 (0.6–15.2)	2.2 (0.8–6.2)	1.2 (0.3–5.3)	0.4 (0.04–4.9)	1.8 (0.9–3.8)
Makeshift	2.7 (0.6–12.2)	1.9 (0.7–5.0)	2.7 (0.8–9.1)	2.1 (0.4–9.6)	2.3 (1.2–4.4)*
Age (years)					
1–4 (referent)	1.0	1.0	1.0	1.0	1.0
5–10	0.2 (0.05–0.9)*	1.0 (0.4–2.1)	1.1 (0.3–3.6)	1.3 (0.3–5.3)	0.9 (0.5-1.5)
11–15	1.3 (0.5–3.2)	1.9 (0.9–4.2)	4.1 (1.5–11.3)**	3.0 (0.8–11.3)	2.5 (1.5–4.1)**
How long in Bangladesh					
1–7 months	NA	NA	NA	NA	NA
8–10 months (referent)	1.0	1.0	1.0	1.0	1.0
> 10 months	0.8 (0.3–1.8)	0.8 (0.4–1.4)	0.5 (0.2–1.1)	0.2 (0.04–0.9)*	0.7 (0.39–0.95)*

* Indicates Statistical Significance of p < 0.05

** Indicates Statistical Significance of p < 0.01

## Discussion

In this study, blood samples were assayed from children residing in three refugee settlements in southeast Bangladesh for *Plasmodium* antigens as well as IgG antibodies against malaria. Presence of the pan-*Plasmodium* antigen aldolase and *P. falciparum* HRP2 antigens have both been shown to be indicative of active infection [19], but HRP2 can potentially linger in blood circulation for months following resolution of infection [24]. Since no children’s samples were found to be antigen positive, the data presented here provide strong evidence that there was no active malaria infections in the children sampled from these settlements, and for a lack of *P. falciparum* infections within the past few months. The findings of no cases of active malaria infections in this survey are consistent with World Health Organization surveillance in 2018 showing a total of 83 confirmed cases among all FDMN camps among nearly a million persons living in these camps (WHO Epidemiological Bulletin Week 52, 2018).

A proportion of the children of all ages and residing in all settlements were found to have IgG antibodies against all three species assayed for: *P. falciparum, P. vivax,* and *P. malariae.* This finding was not unexpected as this region of the world is known to be co-endemic for these three species, and this region represents higher transmission zones for both Bangladesh and Myanmar [1, 25]. Since the MSP1 antigen has been shown to produce a robust IgG response which is known to potentially persist for years following malaria infection [7], it is not possible to determine the timing of exposure from a single cross-sectional survey. No significant differences were seen in anti-malarial antibody carriage from children among all three settlements for any of the three malaria species. It should be noted that children sampled from the Nayapara camp would have been less than 7 years old, and likely due to the few number of seropositives in all camps, the OR estimates were overlapping for all IgG seroprevalence estimates among the three sites. Even when analysis in comparing the three camps was restricted only to persons under 7 years, prevalence and OR estimates were still overlapping. In total, these settlement populations are likely a mixture of persons coming from different malaria transmission settings (and different exposure histories) as indicated by the non-significant OR estimates for all comparisons and wide confidence intervals among the three camps (Table 2). Antibody positivity of a sample to any of the three *P. falciparum*-specific short-term antigens (LSA1, CSP, GLURP-R0) gave strong evidence for a more recent exposure to *P. falciparum* malaria. Overall, low numbers of children were found to be positive for these short-term markers of *P. falciparum* exposure, and no significant differences were again seen in IgG prevalence to these *P. falciparum* markers among settlements.

Correlation of positivity to IgG among the different malaria antigens is displayed in Additional file 1. The prevalence of IgG antibodies against the long term MSP1 antigens was found to be higher among older children in our study, which is expected due to their increased span of lifetime to potentially be exposed to malaria parasites [26]. This was observed for the PmMSP1, PvMSP1, and PfMSP1 antigens, with the 11–14 age category showing increased ORs of 1.3, 1.9, and 4.1 (respectively) when compared to the youngest age category of 1–5 (though the PfMSP1 OR was the only statistically significant increase). Even among children of different ages, differences in prevalence of IgG to MSP1 antigen have been observed previously [27], indicating antibodies against this antigen can provide great resolution with respect to malaria exposure of young persons. Antibody positivity to any of the three short-term *P. falciparum* antigens was also found to increase with increasing age, but with low overall seroprevalence (1.0% of all children), these increases were not statistically significant. Overall, seropositivity for IgG against any *Plasmodium* antigens was found to be significantly higher in the 11–14 age category when compared to the younger categories.

Regardless of age or camp, no children residing in Bangladeshi settlements for less than 8 months were found to have antibodies against of the short-term malaria antigen targets. Additionally, children residing in settlements for longer than 10 months were found to have lower likelihood of IgG seropositivity to all antigens tested when compared with children residing in settlements for 8–10 months. In comparing these two durations of residence (8–10 months versus > 10 months), significant differences were seen for short-term *P. falciparum* antibodies (1.7% versus 0.3%) and antibodies to any malaria (8.9% versus 5.6%). Since the survey of the refugee settlements took place from April to May 2018, children travelling to settlements 8–10 months prior to this would have been in transit from July to September 2017, which is directly in the middle of the monsoon season for this region. Monsoon season in Bangladesh is from June to October [28], in which this region would receive significantly more rain in comparison to the dry season [29]. The peak of monsoon season (and creation of mosquito breeding habitats) for Cox’s Bazar occurs June–August [30], and this Bangladeshi/Myanmar border region is known for high malaria burden for both of these nations [12]. Six *Anopheline* species have been identified as vectors for *Plasmodium* in Bangladesh, and the period of peak transmittance corresponds directly to the monsoon season. Being in transit from Myanmar to the refugee settlements during the peak monsoon and malaria transmittance period could potentially render a mobile population more susceptible to malaria exposure during their time of passage. Children residing in the Bangladeshi settlements longer than 10 months would simulate a counterfactual population to compare malaria exposure since the monsoon still occurred in the refugee settlements (and mosquito breeding habitats still created), but it appears likelihood of malaria exposure was reduced for children living in these settlements with most pronounced differences for short-term markers of *P. falciparum*. Higher risk of malaria exposure during transit could be from a variety of factors including a lack of personal protection from mosquitoes during the monsoon season, or that during transit the refugees may not have had access to stable, consistent shelter. In contrast, residence in these three Bangladeshi refugee settlements appears to have a protective effect with respect to malaria exposure.

## Conclusions

Through serological testing of malaria antigen and anti-malaria IgG antibodies, active transmission of malaria does not appear to be a major threat in the Kutupalong Registered Camp, Nayapara Registered Camp, and Kutupalong Makeshift Settlements in Cox’s Bazar, Bangladesh. None of the 1239 children’s samples was found to be positive for malaria antigens, providing strong evidence for lack of active malaria infections at the time of sampling. Additionally, as the time spent in Bangladesh increases, the likelihood of antibody seropositivity to malaria species *P. falciparum, P. vivax,* and *P. malariae* is reduced with the strongest decreases in short-lived anti-*P. falciparum* IgG. Overall, multiplex bead assay has provided a simplified approach for testing current malaria parasite infection status and past exposure history in a refugee population and ruled out malaria as a major risk factor in their current settlement.

## Supplementary information

**Additional file 1.** Correlation of positivity for IgG against different malaria antigens.

## Data Availability

All data is available upon reasonable request.

## Abbreviations

BSA: Bovine serum albumin
CSP: Circumsporozoite protein
DBS: Dried blood spot
GLURP-R0: Glutamate rich protein, R0 fragment
IgG: Immunoglobulin G
LSA1: Liver stage antigen 1
MSP1: Merozoite surface protein 1
PBS: Phosphate buffered saline

## References

[1] WHO. World malaria report 2017. Geneva: World Health Organization; 2017. https://www.who.int/malaria/publications/world-malaria-report-2017/en/. Accessed 31 July 2019.

[2] Malaria’s Impact Worldwide. https://www.cdc.gov/malaria/malaria_worldwide/impact.html. Accessed 31 July 2019.

[3] Malaria mortality among children under five is concentrated in sub-Saharan Africa. https://data.unicef.org/topic/child-health/malaria/. Accessed 31 July 2019.

[4] Druilhe P, Perignon JL (1994). Mechanisms of defense against *P. falciparum* asexual blood stages in humans. Immunol Lett..

[5] Cutts JC, Powell R, Agius PA, Beeson JG, Simpson JA, Fowkes FJ (2014). Immunological markers of *Plasmodium vivax* exposure and immunity: a systematic review and meta-analysis. BMC Med..

[6] Cherif MK, Ouedraogo O, Sanou GS, Diarra A, Ouedraogo A, Tiono A (2017). Antibody responses to *P. falciparum* blood stage antigens and incidence of clinical malaria in children living in endemic area in Burkina Faso. BMC Res Notes..

[7] Ondigo BN, Hodges JS, Ireland KF, Magak NG, Lanar DE (2014). Estimation of recent and long-term malaria transmission in a population by antibody testing to multiple *Plasmodium falciparum* antigens. J Infect Dis..

[8] Priest JW, Plucinski MM, Huber CS, Rogier E, Mao B, Gregory CJ (2018). Specificity of the IgG antibody response to *Plasmodium falciparum, Plasmodium vivax,* P*lasmodium malariae,* and *Plasmodium ovale* MSP119 subunit proteins in multiplexed serologic assays. Malar J..

[9] White MT, Griffin JT, Akpogheneta O, Conway DJ, Koram KA, Riley EM (2014). Dynamics of the antibody response to *Plasmodium falciparum* infection in African children. J Infect Dis.

[10] Akpogheneta OJ, Duah NO, Tetteh KK, Dunyo S, Lanar DE, Pinder M (2008). Duration of naturally acquired antibody responses to blood-stage *Plasmodium falciparum* is age dependent and antigen specific. Infect Immun.

[11] Alam MS, Khan MGM, Chaudhury N, Deloer S, Nazib F, Bangali AM (2010). Prevalence of anopheline species and their *Plasmodium* infection status in epidemic-prone border areas of Bangladesh. Malar J..

[12] Haque U, Overgaard HJ, Clements ACA, Norris DE, Islam N, Karim J (2014). Malaria burden and control in Bangladesh and prospects for elimination: an epidemiological and economic assessment. Lancet Global Health.

[13] Rahman M, Rahman R, Bangali M, Das S, Talukder MR, Ringwald P (2004). Efficacy of combined chloroquine and sulfadoxine–pyrimethamine in uncomplicated *Plasmodium falciparum* malaria in Bangladesh. Trans R Soc Trop Med Hyg.

[14] Group ISC. Situation Report: Rohingya Refugee Crisis. 2018.

[15] Rohingya Refugee Crisis. https://www.unocha.org/rohingya-refugee-crisis. Accessed 31 July 2019.

[16] Cousins S (2018). Rohingya threatened by infectious diseases. Lancet Infect Dis..

[17] Keeping a close watch to prevent malaria outbreak in Cox’s Bazar. https://reliefweb.int/report/bangladesh/keeping-close-watch-prevent-malaria-outbreak-cox-s-bazar. Accessed 31 July 2019.

[18] ISCG. Situation report: Rohingya refugee crisis. United Nations Office for the Coordination of Humanitarian Affairs. 2018.

[19] Plucinski MM, Herman C, Jones S, Dimbu R, Fortes F, Ljolje D (2018). Screening for Pfhrp2/3-deleted *Plasmodium falciparum*, non-falciparum, and low-density malaria infections by a multiplex antigen assay. J Infect Dis.

[20] Plucinski MM, Candrinho B, Chambe G, Muchanga J, Muguande O, Matsinhe G (2018). Multiplex serology for impact evaluation of bed net distribution on burden of lymphatic filariasis and four species of human malaria in northern Mozambique. PLoS Negl Trop Dis..

[21] Kerkhof K, Canier L, Kim S, Heng S, Sochantha T, Sovannaroth S (2015). Implementation and application of a multiplex assay to detect malaria-specific antibodies: a promising tool for assessing malaria transmission in Southeast Asian pre-elimination areas. Malar J..

[22] Rogier E, Moss DM, Chard AN, Trinies V, Doumbia S, Freeman MC (2017). Evaluation of immunoglobulin G responses to *Plasmodium falciparum* and *Plasmodium vivax* in Malian school children using multiplex bead assay. Am J Trop Med Hyg.

[23] Scobie HM, Mao B, Buth S, Wannemuehler KA, Sorensen C, Kannarath C (2016). Tetanus immunity among women aged 15 to 39 years in Cambodia: a national population-based serosurvey, 2012. Clin Vaccine Immunol.

[24] Plucinski MM, Dimbu PR, Fortes F, Abdulla S, Ahmed S, Gutman J (2018). Posttreatment HRP2 clearance in patients with uncomplicated *Plasmodium falciparum* malaria. J Infect Dis.

[25] Ahmed SM, Hossain S, Kabir MM, Roy SJMJ (2011). Free distribution of insecticidal bed nets improves possession and preferential use by households and is equitable: findings from two cross-sectional surveys in thirteen malaria endemic districts of Bangladesh. Malar J..

[26] Stanisic DI, Fowkes FJI, Koinari M, Javati S, Lin E, Kiniboro B (2015). Acquisition of antibodies against *Plasmodium falciparum* merozoites and malaria immunity in young children and the influence of age, force of infection, and magnitude of response. Infect Immun.

[27] Omosun YO, Anumudu CI, Adoro S, Odaibo AB, Sodeinde O, Holder AA (2005). Variation in the relationship between anti-MSP-1(19) antibody response and age in children infected with *Plasmodium falciparum* during the dry and rainy seasons. Acta Trop.

[28] Ahmed R, Karmakar S (1993). Arrival and withdrawal dates of the summer monsoon in Bangladesh. Royal Meterol Soc..

[29] Burkart K, Kinney P (2016). Is precipitation a predictor of mortality in Bangladesh? A multi-stratified analysis in a South Asian monsoon climate. Sci Total Environ.

[30] Climate Cox’s Bazar. https://en.climate-data.org/asia/bangladesh/chittagong-division/cox-s-bazar-56253/#climate-graph. Accessed 31 July 2019.

