# Supplementary material for: Screening for malaria antigen and anti-malarial IgG antibody in forcibly-displaced Myanmar nationals: Cox’s Bazar district, Bangladesh, 2018
Source: Malar J. 2020 Mar 30;19:130. doi: 10.1186/s12936-020-03199-4 (PMC7106647; doi:10.1186/s12936-020-03199-4)
Supplement: Supplementary file 1 — Additional file 1. Correlation of positivity for IgG against different malaria antigens. [file 12936_2020_3199_MOESM1_ESM.docx]

**Additional File 1. Correlation of positivity for IgG against different malaria antigens.**

|  |  | **PfLSA1** | **PfCSP** | **PfGLURP** | **PfMSP1** | **PmMSP1** | **PvMSP1** |
| --- | --- | --- | --- | --- | --- | --- | --- |
| Pearson Correlation Coefficient | **PfLSA1** | 1 | -0.02833 | -0.02833 | -0.06756 | -0.06029 | -0.09269 |
| p value (at alpha 0.05) |  |  | 0.791 | 0.791 | 0.5269 | 0.5724 | 0.3849 |
|  | **PfCSP** | -0.02833 | 1 | -0.07143 | -0.07207 | -0.15202 | -0.23371 |
|  |  | 0.791 |  | 0.5035 | 0.4997 | 0.1526 | 0.0266 |
|  | **PfGLURP** | -0.02833 | -0.07143 | 1 | -0.17035 | -0.15202 | -0.23371 |
|  |  | 0.791 | 0.5035 |  | 0.1084 | 0.1526 | 0.0266 |
|  | **PfMSP1** | -0.06756 | -0.07207 | -0.17035 | 1 | -0.07732 | -0.40896 |
|  |  | 0.5269 | 0.4997 | 0.1084 |  | 0.4688 | <.0001 |
|  | **PmMSP1** | -0.06029 | -0.15202 | -0.15202 | -0.07732 | 1 | -0.39305 |
|  |  | 0.5724 | 0.1526 | 0.1526 | 0.4688 |  | 0.0001 |
|  | **PvMSP1** | -0.09269 | -0.23371 | -0.23371 | -0.40896 | -0.39305 | 1 |
|  |  | 0.3849 | 0.0266 | 0.0266 | <.0001 | 0.0001 |  |
